# Supplementary figures and images for: Benchmarking a highly selective USP30 inhibitor for enhancement of mitophagy and pexophagy
Source: Life Sci Alliance. 2021 Nov 29;5(2):e202101287. doi: 10.26508/lsa.202101287 (PMC8645336; doi:10.26508/lsa.202101287)

Source Data: Figure 1C

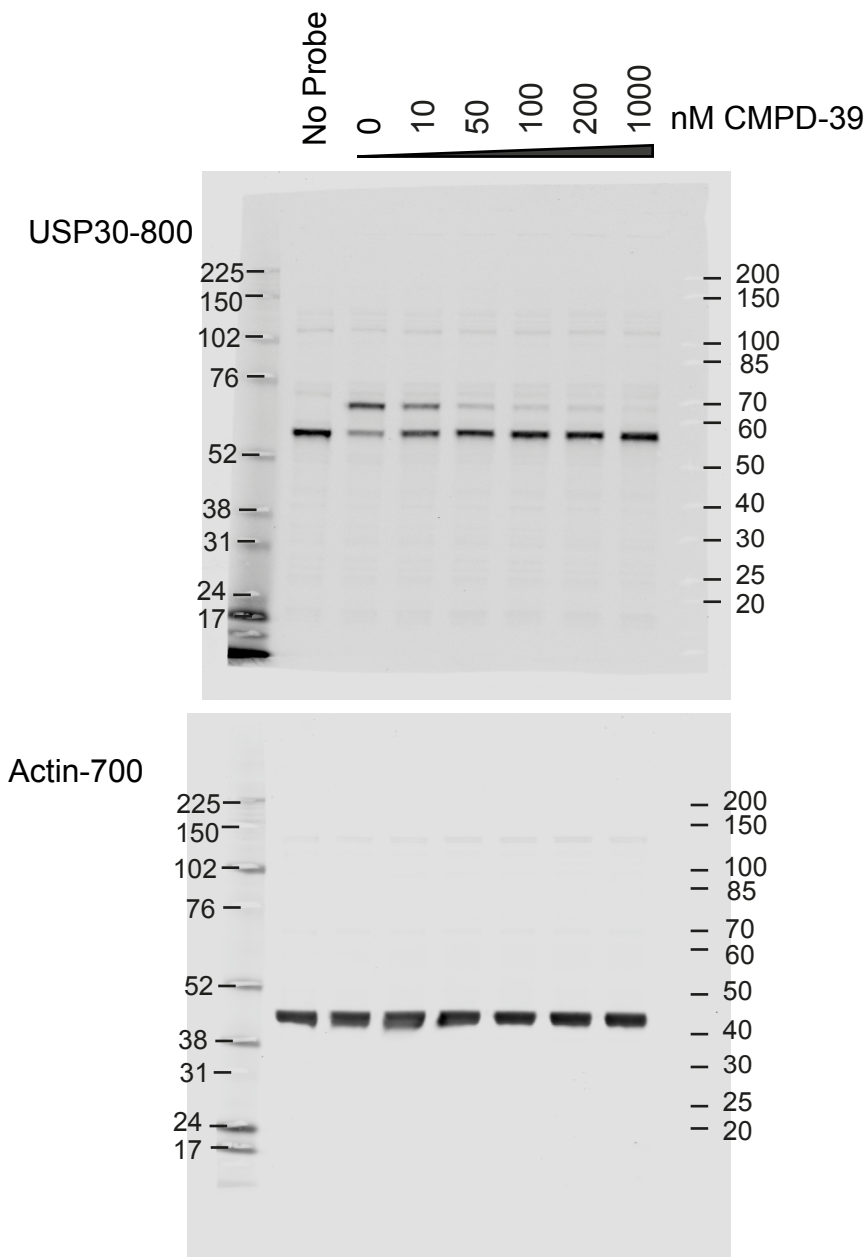

Source Data: Figure 1D

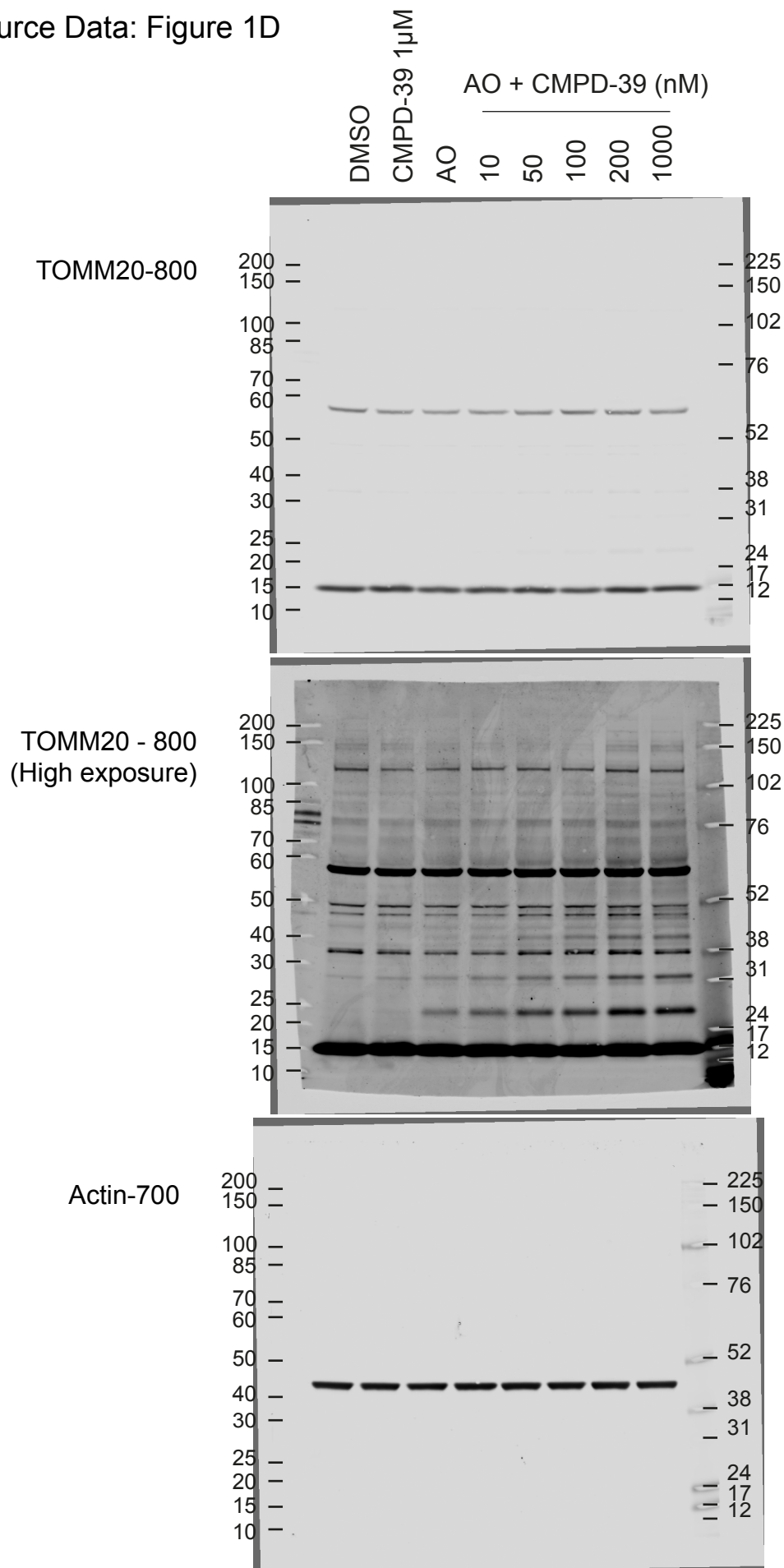

Supplement: Supplementary file 3 [file LSA-2021-01287_SdataF2.2.pdf]
