# Supplementary material for: Benchmarking a highly selective USP30 inhibitor for enhancement of mitophagy and pexophagy
Source: Life Sci Alliance. 2021 Nov 29;5(2):e202101287. doi: 10.26508/lsa.202101287 (PMC8645336; doi:10.26508/lsa.202101287)

Source Data: Figure 2A

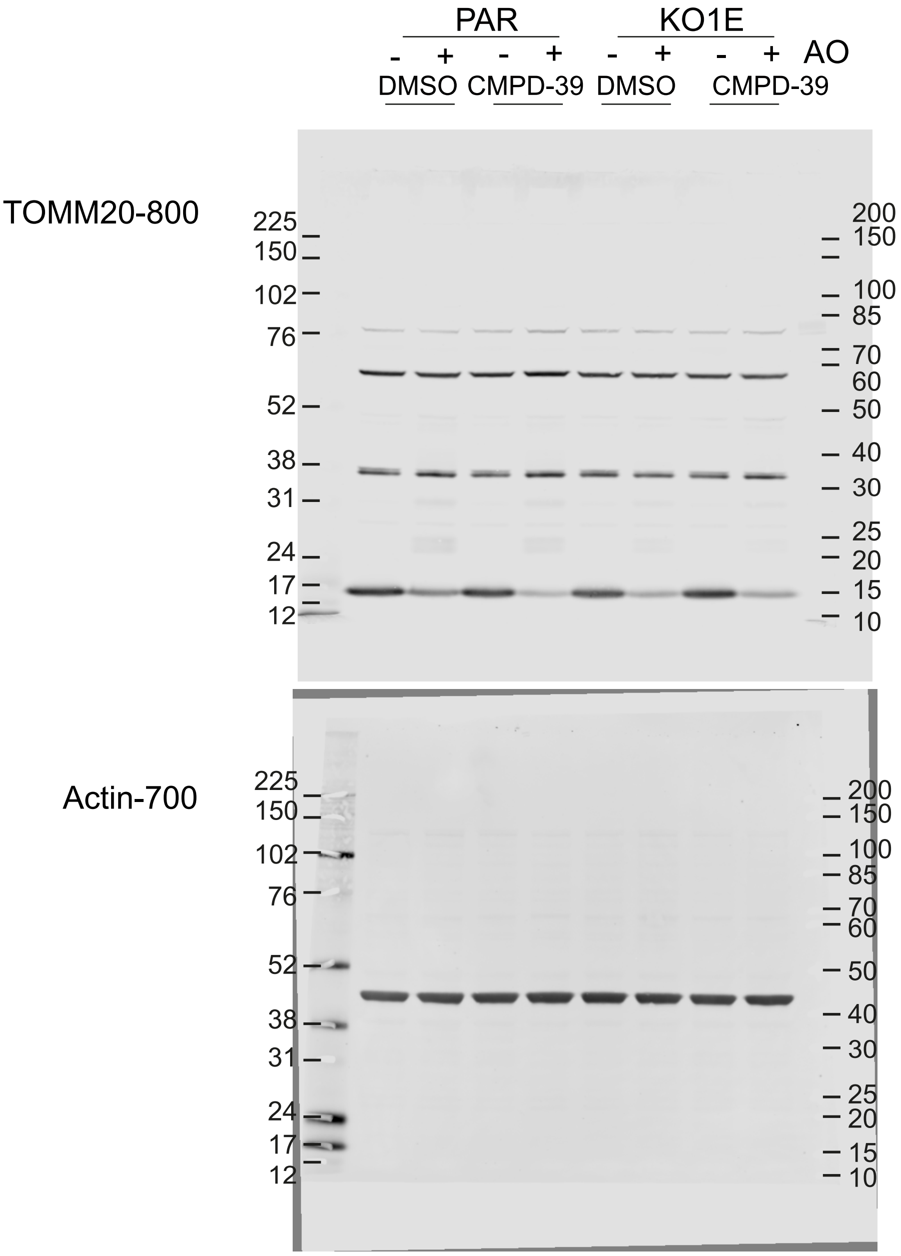

Source Data: Figure 2B
Blot1

TOM20-800

TOMM20 - 800
(High exposure)

Actin-700

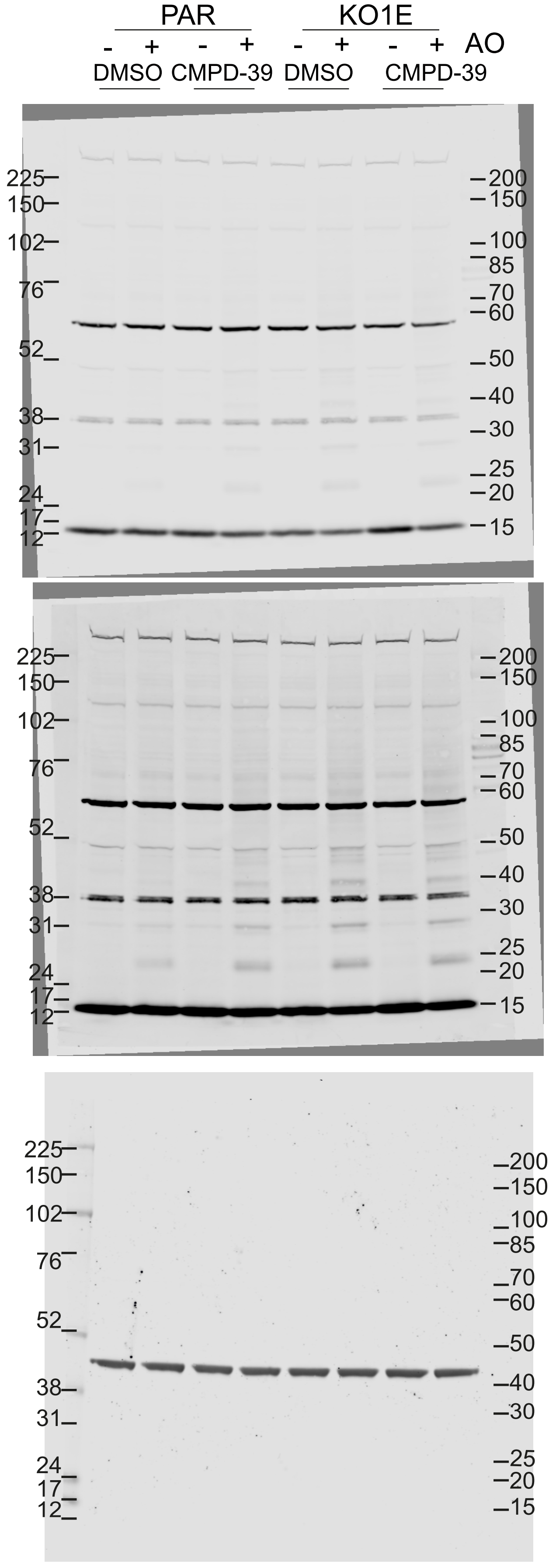

Source Data: Figure 2B

Blot2

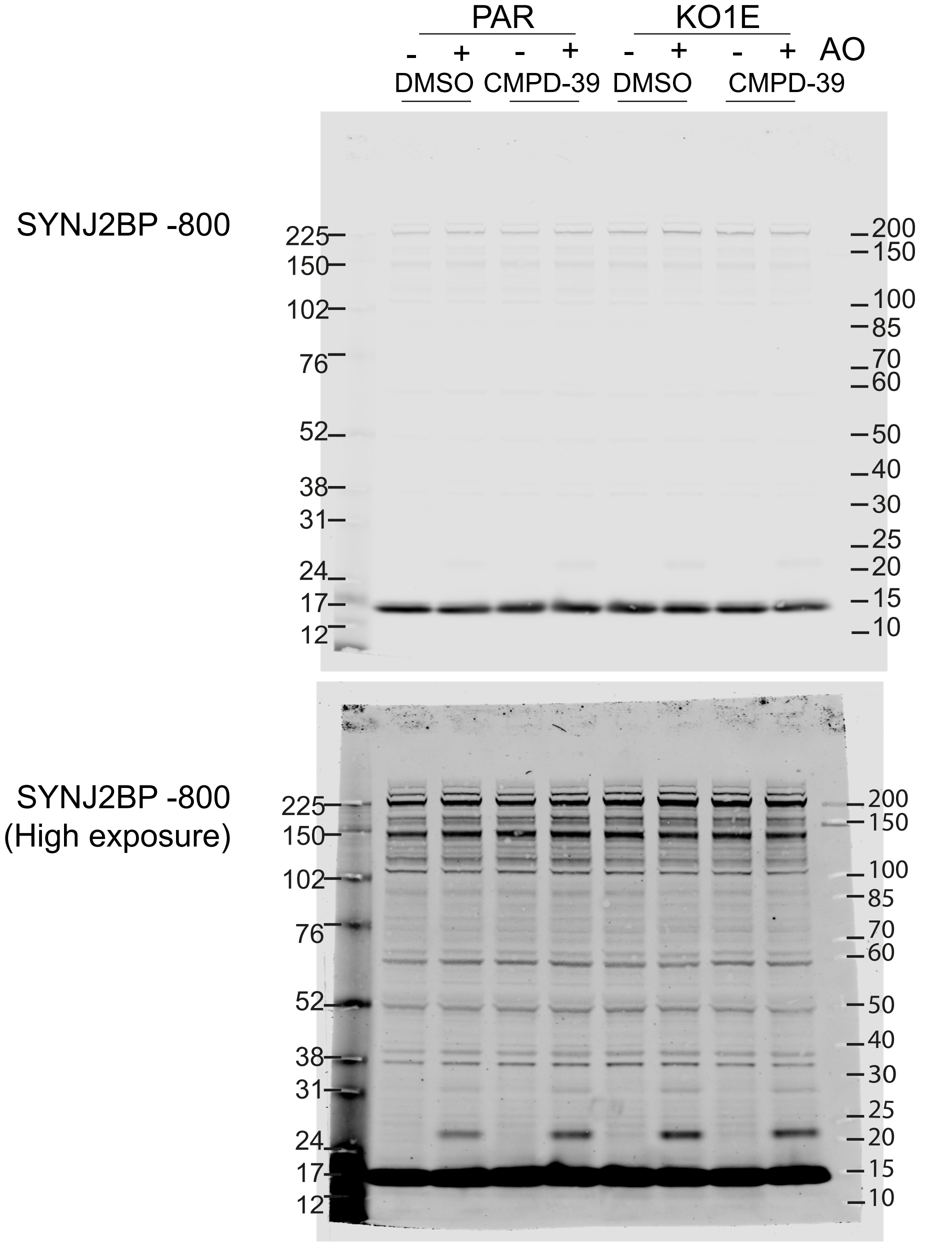

Source Data: Figure 2B

Blot2 Cont.

Reprobed for USP30 - 800
(After SYNJ2BP)

Actin-700

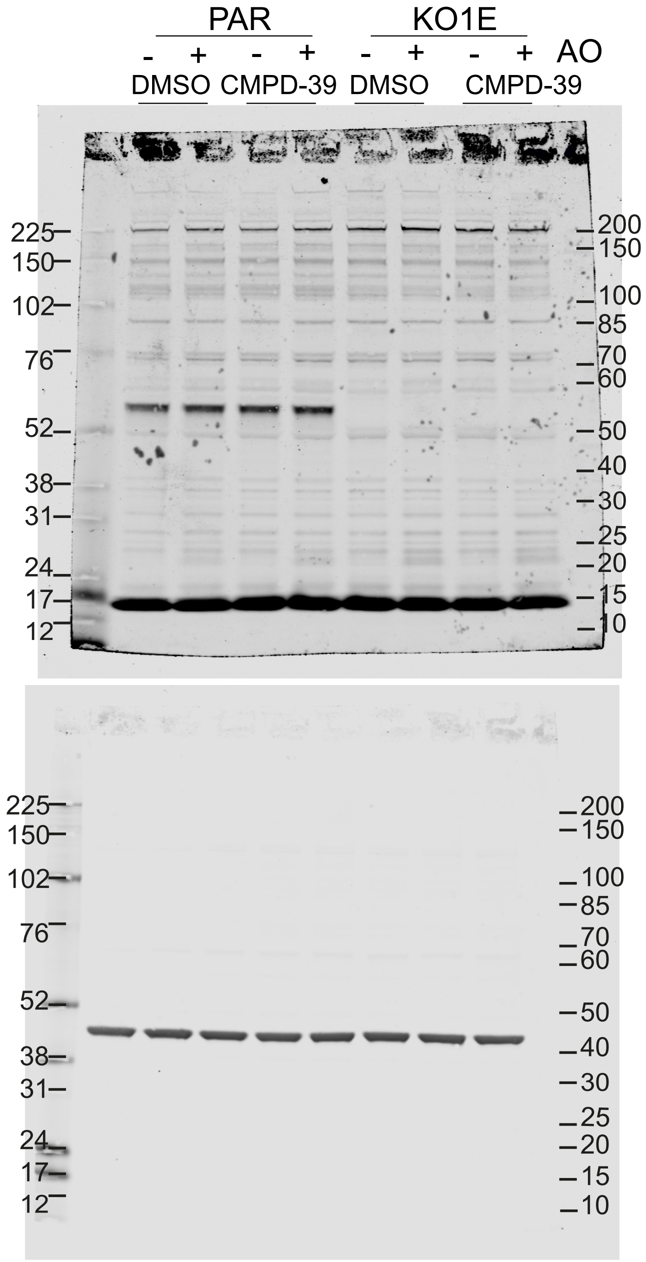

Source Data: Figure 2E

Blot1

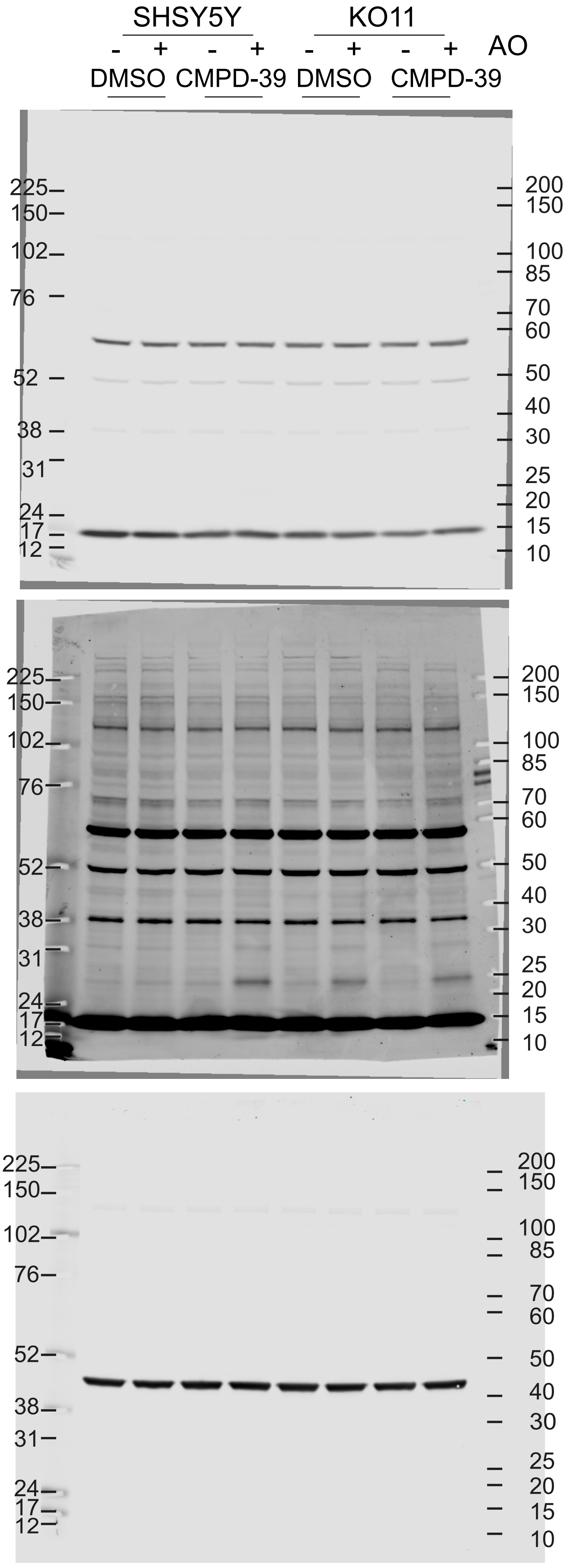

TOM20-800

TOMM20 - 800
(High exposure)

Actin-700

Source Data: Figure 2E

Blot2

SYNJ2BP -800

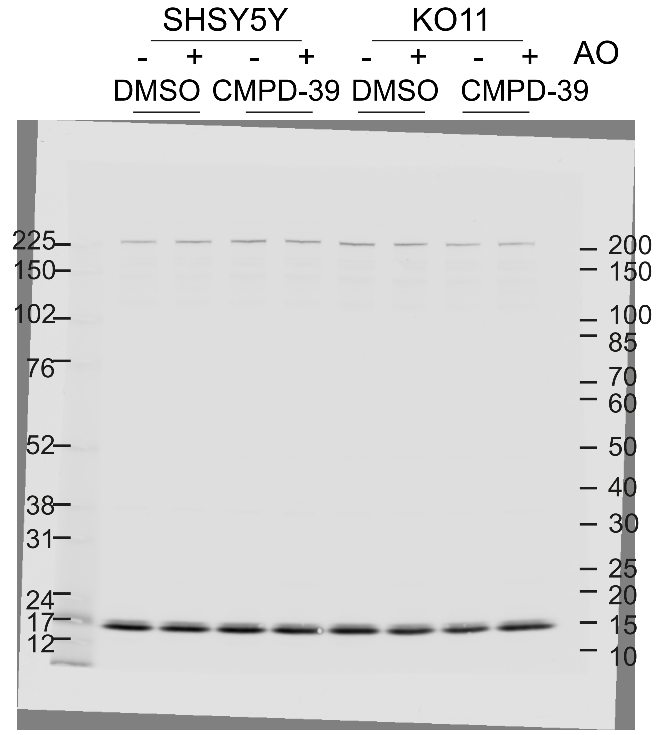

SYNJ2BP -800
(High exposure)

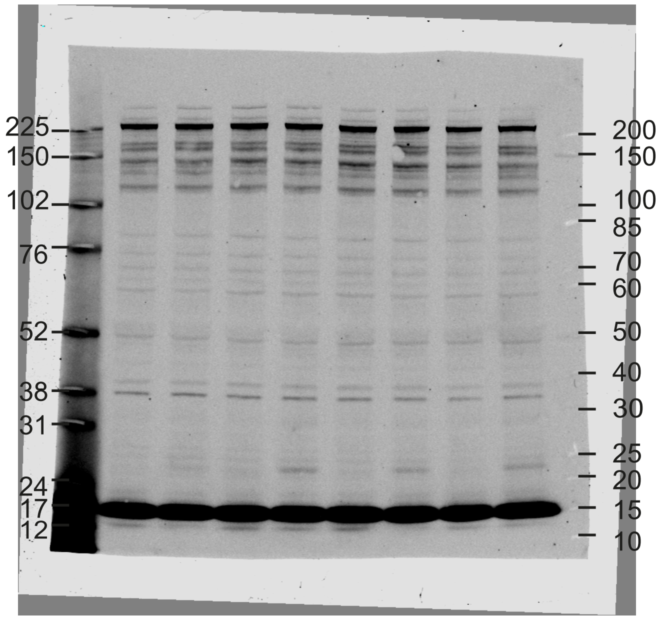

Source Data: Figure 2E

Blot2 Cont.

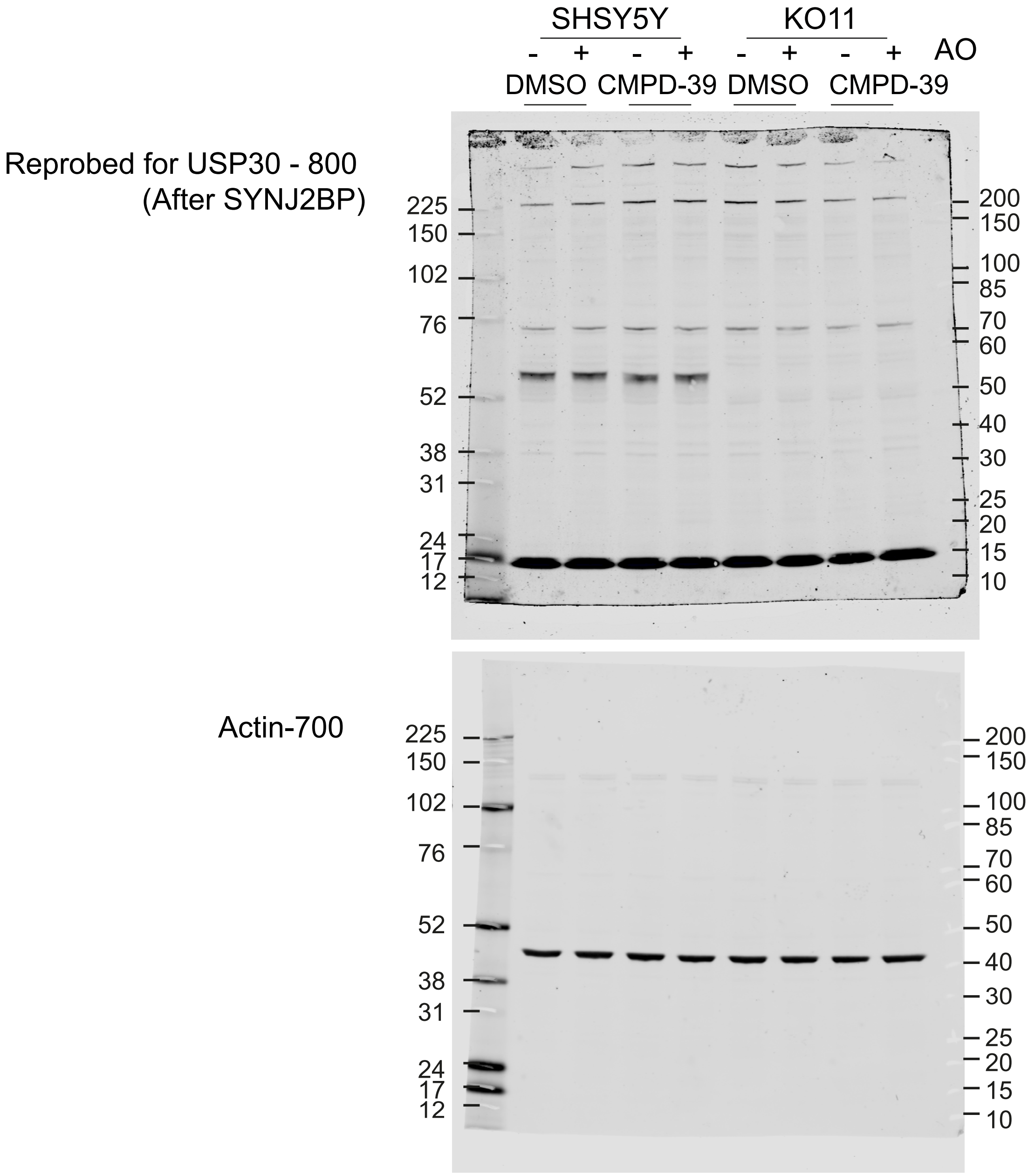

Source Data: Figure 2E

Blot3

PhosphoUb-S65-800

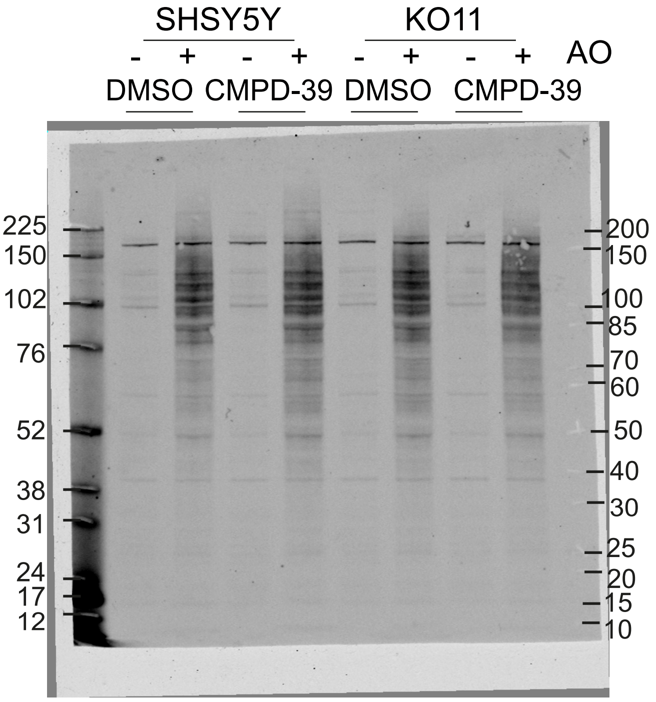

Actin-700

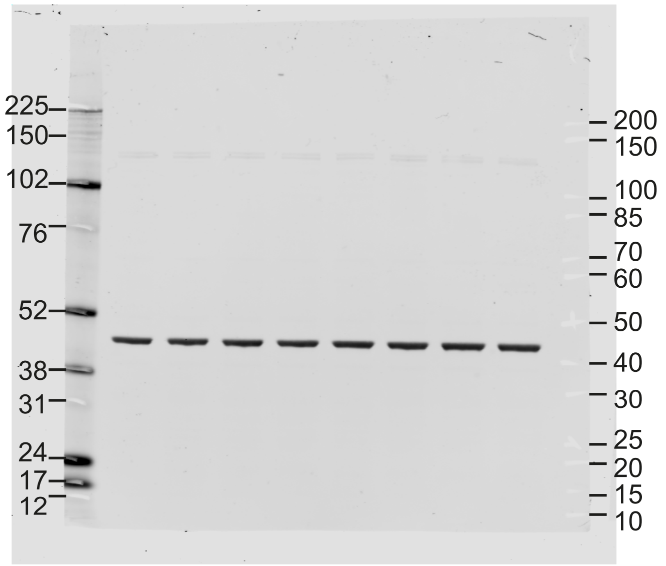

Supplement: Supplementary file 4 [file LSA-2021-01287_SdataF2.3.pdf]
